# *In vivo* discovery of blood-brain barrier opening small molecules with FishNAP

**DOI:** 10.64898/2026.03.18.712473

**Published:** 2026-03-20

**Authors:** Thomas C. Potts, Erin E. McDonnell, Lewis G. Gall, Evangeline Coffinas, Jacklyn Levey, Colleen G. Rutley, Neha Gururaj, Alexandra K. Vindigni, Advait R. Iyer, Manan H. Gosai, Megan E. Canace, Natasha M. O’Brown

**Affiliations:** 1 Department of Cell Biology and Neuroscience, Rutgers University, Piscataway, NJ 08854, USA; 2 Howard Hughes Medical Institute, Piscataway, NJ 08854, USA

## Abstract

The blood–brain barrier (BBB) is crucial for neural homeostasis, tightly regulating molecular exchange between the circulation and brain. However, this selective protection also greatly limits drug delivery to the central nervous system, posing a major challenge for treating neurological disorders. Pharmacological strategies that transiently and safely increase BBB permeability could therefore transform brain drug delivery, yet systematic discovery of such modulators remains hampered by the limitations of current *in vitro* and *in vivo* approaches. Here we present FishNAP, a non-invasive, high-throughput zebrafish platform for real-time assessment of BBB permeability *in vivo*. FishNAP captures developmental changes in barrier function and detects dysfunction in genetic mutants. Using this platform, we screened 2,320 FDA-approved small molecules for compounds capable of opening an intact BBB and identified 11 that reproducibly increased permeability. Seven of these molecules allowed entry of a 1 kDa tracer into brain tissue, and five also permitted passage of a larger 10 kDa Dextran. Barrier integrity recovered within 24 hours for all seven compounds, indicating reversible modulation. Finally, testing three representative molecules (Calcitriol, Lovastatin, and Sunitinib) in adult mice revealed increased BBB permeability and reduced Claudin- 5 expression, demonstrating conserved mechanisms of BBB-regulation across vertebrates. FishNAP thus enables systematic discovery of BBB modulators with direct translational potential for brain drug delivery.

## Introduction

The blood–brain barrier (BBB) arises from specialized properties of the brain vasculature that tightly regulate molecular and cellular exchange between the circulation and brain parenchyma. This selective permeability is essential for maintaining the brain’s homeostatic environment and protecting neuronal tissue from toxins and pathogens^1^. Barrier function arises from tight junctions between endothelial cells, suppression of vesicular transcytosis, and specialized transport systems that regulate nutrient influx and waste removal^2^. While these features are critical for neural health, they also present a major obstacle for central nervous system (CNS) therapeutics—over 98% of small molecules fail to reach the brain at pharmacologically relevant concentrations^3^. Consequently, substantial effort has focused on strategies to bypass or transiently modulate the BBB, including chemical modification of drugs to enhance penetration, receptor- mediated transport, and focused ultrasound to transiently disrupt tight junctions^4,5^. Although these approaches have shown promise in specific contexts, identifying compounds capable of safely and reversibly modulating the intact BBB remains challenging.

A number of experimental platforms have been developed to study BBB permeability and evaluate candidate therapeutics. *In vitro* systems—including transwell assays, organ-on-chip technologies, and human induced pluripotent stem cell (iPSC)-derived neurovascular units—have provided valuable insight into endothelial barrier biology and offer tractable platforms for mechanistic studies and drug testing in the human context^6,7^. However, these models do not fully capture the complex cellular interactions and physiological forces present in the intact brain vasculature. Indeed, the maintenance of BBB identity depends on signaling between endothelial cells and surrounding cells in the brain, including pericytes, neurons, and astrocytes^8-12^, interactions that are difficult to fully reproduce *in vitro*, resulting in a rapid loss of these BBB molecular signatures^13^. As a result, there remains a need for experimental systems that preserve the physiological context of the BBB while remaining compatible with scalable discovery approaches.

Zebrafish provide an opportunity to bridge this gap. The zebrafish BBB shares key molecular and structural features with the mammalian barrier and becomes functionally restrictive by 5 days post-fertilization (dpf)^1,14,15^. In addition to this evolutionary conservation, zebrafish are highly tractable genetic and chemical systems: their external development, small size, and capacity for direct compound absorption from the surrounding water enable rapid *in vivo* manipulation and screening^16^. However, most existing zebrafish assays for BBB permeability rely on tracer injections or confocal imaging^8,15,17^, which substantially limit experimental throughput.

To address this limitation, we developed FishNAP, a zebrafish-based platform for high-throughput measurement of BBB permeability *in vivo*. FishNAP leverages the small size and optical transparency of zebrafish larvae to measure barrier permeability in a 96-well format using a behavioral phenotype that correlates with BBB leakage. Because this assay does not require tracer injections or high-resolution imaging, it enables rapid and scalable assessment of BBB modulation across large chemical libraries. Using FishNAP, we screened 2,320 FDA-approved small molecules and identified 11 compounds that reproducibly increased BBB permeability. Follow-up tracer leakage assays narrowed this to 7 molecules that robustly opened the zebrafish BBB. Importantly, three compounds (Calcitriol, Lovastatin, and Sunitinib), representing distinct signaling pathways, also increased BBB permeability in adult mice, indicating conservation of barrier modulation across vertebrates. Together, these findings establish FishNAP as a scalable *in vivo* platform for discovering pharmacological modulators of BBB permeability and identify candidate compounds capable of transiently opening the intact barrier.

## Results

### Novel FishNAP assay for BBB function

**Fig. 1.**
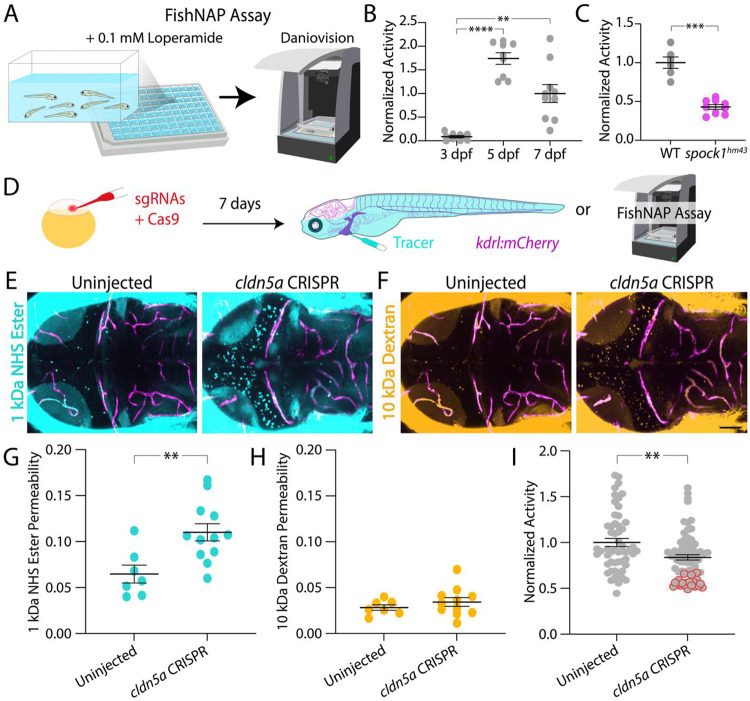
FishNAP reliably reveals increased BBB permeability. **A.** Schematic of the FishNAP assay, with up to seven larvae per well. Overall activity is monitored using Daniovision for 1 hour after loperamide exposure; increased BBB permeability leads to reduced locomotor activity. **B.** Leaky 3 dpf larvae are significantly sedated compared to 5 and 7 dpf fish, as previously reported (O’Brown, 2019). **C.** *spock1* mutants show significantly reduced activity compared to wild-type (WT) controls at 7 dpf using FishNAP. **D.** Workflow schematic for assessing BBB permeability in *cldn5a* crispant fish. **E–H.** Tracer leakage assays using 1 kDa NHS ester (E, turquoise) and 10 kDa Dextran (F, gold) reveal significantly increased 1 kDa NHS tracer permeability outside of the *kdrl:mCherry*+ vasculature (magenta) in *cldn5a* crispants vs. uninjected controls (G), but no significant change in 10 kDa Dextran permeability (H). **I.** FishNAP also detects increased BBB permeability in *cldn5a* crispants relative to uninjected controls. The slowest fish (red outlines) show greater than 95% *cldn5a* knockdown. ** p<0.01, *** p<0.001, **** p<0.0001 by one-way ANOVA (B and C) and Welches t-test (G-I). Scale bar represents 50 μm.

To enable medium-throughput, *in vivo* assessment of BBB function, we developed a scalable behavioral screening platform called FishNAP (**Fish**-based assay for **N**on-invasive **A**ssessment of **P**ermeability). This assay uses larval zebrafish and measures BBB integrity based on loperamide-induced sedation in a 96-well format using Daniovision (Fig. 1A). Loperamide is a synthetic opioid receptor agonist that does not cross the intact BBB. Thus, larvae with a sealed BBB remain behaviorally active after exposure, whereas larvae with a leaky BBB exhibit reduced activity as loperamide enters the brain and induces sedation. We first validated FishNAP across developmental stages with distinct BBB states. At 3 dpf, when elevated endothelial transcytosis renders the BBB functionally immature^15^, larvae exhibited robust loperamide-induced sedation (Fig. 1B). In contrast, at 5 and 7 dpf, when transcytosis is suppressed and the BBB has functionally sealed^15^, larvae remained behaviorally active following loperamide exposure, consistent with an intact barrier. To further validate the assay, we tested the zebrafish *spock1* mutant line, which has regionally restricted BBB permeability in the forebrain and midbrain due to defective tight junctions and elevated transcytosis^8^, and found that, compared to wild-type controls, *spock1* mutants displayed loperamide-induced sedation (Fig. 1C), demonstrating that FishNAP sensitively detects both developmental and genetically induced BBB dysfunction.

We further tested the FishNAP assay on *cldn5a* F0 crispants, which exhibit mosaic disruption of the tight junction gene *cldn5a* that is essential for BBB integrity^18^. BBB function in these fish was assessed using both functional tracer leakage assays and FishNAP (Fig. 1D). Permeability to a small 1 kDa NHS tracer (Fig. 1E) and a larger 10 kDa Dextran tracer (Fig. 1F) revealed a significant increase in 1 kDa NHS Ester tracer permeability (Fig. 1G), but no significant change in 10 kDa Dextran permeability in the *cldn5a* crispants (Fig. 1H), consistent with altered tight junction function in the mosaic *cldn5a* crispants. Traditional fluorescent tracer leakage assays required approximately three hours for injection, circulation, mounting, and confocal imaging (Fig. 1E-H), whereas FishNAP detected significant BBB impairment in more *cldn5a* crispants compared to control-injected siblings within just one hour (Fig. 1I). RT-PCR analysis showed more than 95% *cldn5a* knockdown in the most sedated crispants (red outline), demonstrating that FishNAP can stratify mosaic knockdown efficiency based on functional outcome. Because this assay is nonlethal and does not require phenylthiourea (PTU) treatment, which is typically used to block pigmentation for confocal imaging in wild-type fish, it enables rapid and scalable screening of BBB function. Notably, larvae showing strong BBB phenotypes could be placed directly on the fish system, streamlining the generation of stable mutant lines by allowing early selection and rearing of the most penetrant individuals.

### Identification of BBB-opening small molecules

**Fig. 2.**
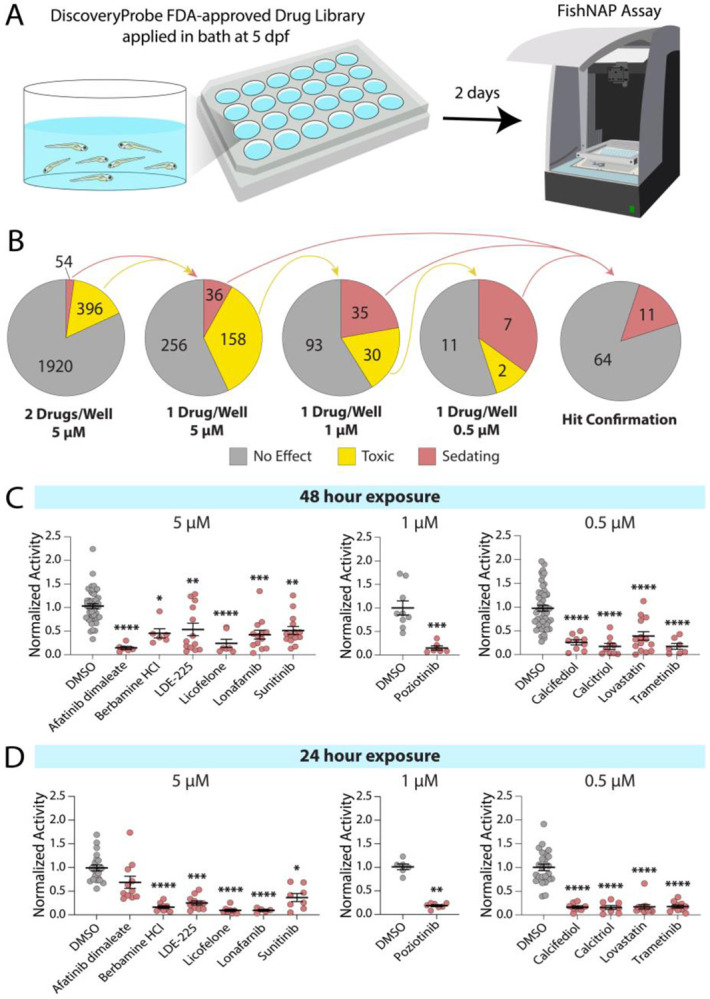
FishNAP-based screening of FDA-approved small molecules identifies 11 compounds that significantly reduce locomotor activity in zebrafish larvae. **A**. Schematic of the small-molecule treatment paradigm in which seven larvae were treated from 5 to 7 dpf in a 24-well plate and assayed for BBB function using FishNAP at 7 dpf. **B**. Pie charts illustrating the screening workflow, beginning with pooled testing at 5 μM, followed by individual compound testing at 5 μM and lower concentrations (1 μM and 0.5 μM) for compounds that were toxic (yellow). Putative hits (puce) were validated by replicate testing, yielding 11 confirmed small-molecule hits. **C**. Validation of the 11 compounds that significantly sedated larvae after 48 hours of treatment compared to DMSO controls at their respective concentrations. **D**. FishNAP reveals that 10 of these compounds can induce sedation even after only 24 hours of treatment. Each point represents an individual well of 7 fish. * p<0.05, ** p<0.01, *** p<0.001, **** p<0.0001 by one-way ANOVA compared to DMSO controls.

Using the FishNAP platform, we screened a library of 2,320 FDA-approved small molecules (ApexBio), to identify compounds capable of opening a sealed BBB. Zebrafish were treated in bath with two pooled compounds, each at 5 μM, from 5 to 7 dpf, and BBB integrity was assessed using FishNAP (Fig. 2A). Putative hits were defined as having an activity ratio below 0.6. Following pooled testing, we deconvoluted the pools by individually testing compounds from toxic or hit pairs. This yielded 36 molecules that induced sedation alone, as well as 158 molecules that remained toxic at 5 μM (Fig. 2B). These 158 toxic molecules were then retested at 1 μM, which revealed an additional 35 compounds capable of inducing sedation at the lower dose and 30 compounds that were still toxic at 1 μM. We then retested these toxic molecules at 0.5 μM, which revealed another 7 small molecules that reduced activity below our 0.6 threshold. All 78 putative hits were then reviewed, and only those with reasonable potential for systemic treatment in humans were selected for follow-up testing. Compounds intended solely for topical use, antiseptics, or food additives were excluded. The remaining candidates were tested in at least three biological replicates to confirm reproducibility, resulting in 11 small molecules that consistently caused BBB leakage in the FishNAP assay after 48 hours of treatment (Fig. 2C). To assess the kinetics of BBB disruption, we tested these 11 molecules after just 24 hours of exposure. We found that 10 of the 11 compounds (all except for Afatinib dimaleate) induced sedation within the shorter time window (Fig. 2D). Together, these results demonstrate the power of FishNAP for *in vivo* screening of BBB-modulating small molecules.

**Fig. 3.**
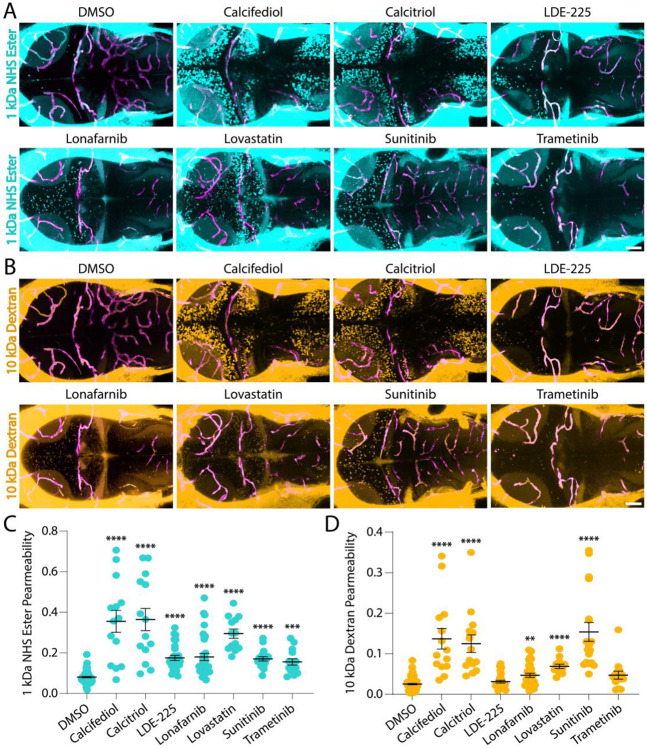
Functional tracer leakage assays validate small molecule opening of the BBB. **A-B.** At 7 dpf, larvae were treated with each small molecule from 5–7 dpf and assayed for leakage of either 1 kDa NHS Ester (turquoise, A) or 10 kDa Dextran (gold, B) from the vasculature (magenta) into the brain, revealing increased permeability compared to DMSO controls. **C–D**. Quantification of 1 kDa NHS Ester (C) and 10 kDa Dextran (D) tracer permeability reveals that all 7 molecules significantly increase permeability to the small 1 kDa tracer, with varying degrees of leakage of the 10 kDa tracer. Specifically, Calcifediol, Calcitriol, and Sunitinib increased BBB permeability to the larger tracer the most, suggesting some transcytosis defects, whereas LDE-225 and Trametinib did not exhibit any leakage of the 10 kDa tracer, suggesting tight junction–specific defects. Each point represents an individual fish. ** p<0.01, *** p<0.001, **** p<0.0001 by one-way ANOVA compared to DMSO controls. Scale bars represent 50 μm.

To confirm that the 11 identified compounds caused sedation through increased BBB permeability rather than off-target effects, we repeated the 48-hour treatment paradigm followed by functional tracer leakage assays. We used two molecular weight tracers, 1 kDa NHS Ester and 10 kDa Dextran, to determine not only whether the BBB was compromised but also the likely subcellular mechanism, since tight junction defects selectively increase permeability to the 1 kDa tracer^19^ , as seen in the *cldn5a* crispants (Fig. 1E-H) whereas elevated transcytosis increases permeability to both^15^. These assays revealed that 8 of the 11 compounds significantly increased BBB permeability compared with DMSO-treated controls (Fig. 3, Fig. S1), and seven—Calcifediol, Calcitriol, LDE-225, Lonafarnib, Lovastatin, Sunitinib, and Trametinib—caused substantial increases in 1 kDa NHS Ester permeability (Fig. 3C). Most of these compounds also enhanced permeability to the larger 10 kDa Dextran tracer, except for LDE-225 and Trametinib, which likely act exclusively through tight junction disruption rather than increased transcytosis (Fig. 3D). Calcitriol, Calcifediol, and Sunitinib produced the greatest increases in 10 kDa Dextran permeability, while Lovastatin and Lonafarnib induced smaller but significant effects. These hits targeted diverse signaling pathways, including Hedgehog (LDE-225), farnesyltransferase/Ras (Lonafarnib), HMG-CoA reductase/cholesterol (Lovastatin), multi-kinase RTK/VEGF (Sunitinib), and MEK/ERK MAPK (Trametinib), with vitamin D signaling (Calcifediol and Calcitriol) as the only pathway with multiple hits. Notably, both vitamin D agonists increased BBB permeability throughout the brain, implicating vitamin D in BBB opening for the first time.

### Recovery of BBB integrity after drug-induced opening

**Fig. 4.**
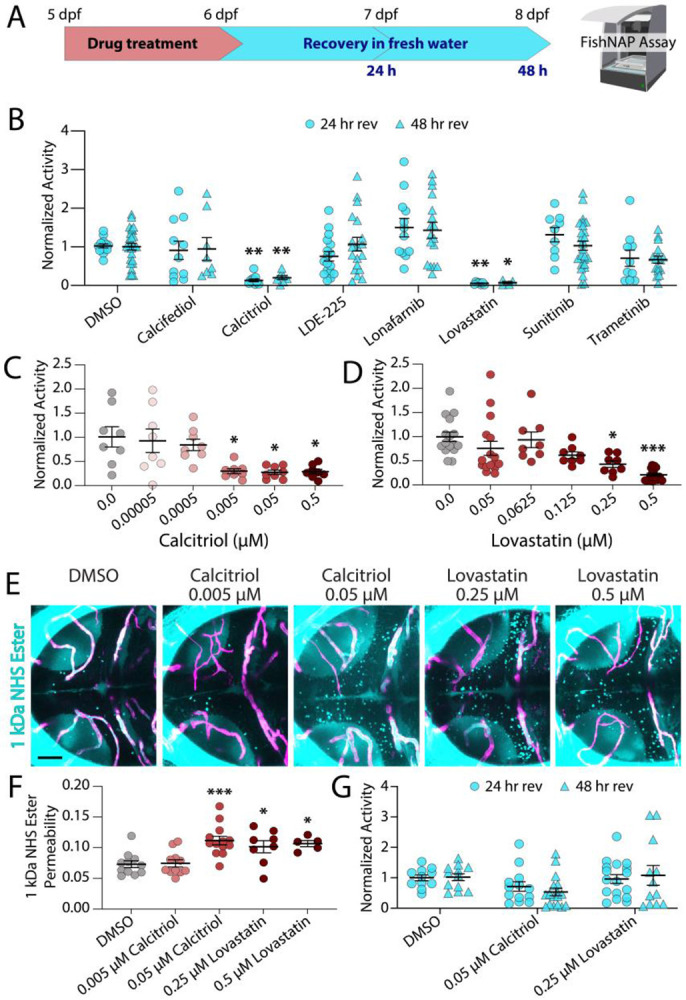
BBB modulation by these seven small molecules is transient. **A.** Schematic of small molecule treatment and recovery paradigm. **B.** Recovery assays reveal that five of the BBB-opening small molecules are able to recover full BBB function within 24 hours (Calcifediol, LDE-225, Lonafarnib, Sunitinib, and Trametinib) at the screening concentrations, while Calcitriol and Lovastatin remain leaky for 48 hours after 0.5 μM exposures. **C-D.** Dose-response curves for Calcitriol (C) and Lovastatin (D) reveal Calcitriol is able to significantly sedate at 0.005 μM and Lovastatin is able to sedate at 0.25 μM. **E-F.** Functional tracer leakage assays with 1 kDa NHS Ester (turquoise, E) reveal 0.05 μM Calcitriol exposure and 0.25 μM Lovastatin exposure are able to significantly increase BBB permeability outside of the vasculature (magenta), while 0.005 μM Calcitriol exposure does not have a significant effect (F). **G.** Recovery assays at lower doses of Calcitriol and Lovastatin reveal BBB functional recovery within 24 hours. Each point represents an individual well of 7 fish (B-D, G) or an individual fish (F). * p<0.05, ** p<0.01, *** p<0.001 by one-way ANOVA compared to DMSO controls. Scale bar represents 50 μm.

To assess whether the effects of these seven BBB-modulating small molecules were reversible, we treated larvae with each compound for 24 hours, which was sufficient to increase BBB permeability (Fig. 2D), then washed into fresh, drug-free media and monitored daily using FishNAP to determine when BBB integrity was restored (Fig. 4A). This revealed full recovery within 24 hours for Calcifediol, LDE-225, Lonafarnib, Sunitinib, and Trametinib (Fig. 4B). However, Calcitriol and Lovastatin at the screening concentration of 0.5 μM failed to recover within 48 hours (Fig. 4B), suggesting either irreversible BBB disruption or prolonged drug retention that maintains pharmacological activity during washout. We therefore determined minimal effective doses to open the BBB within 24 hours using FishNAP, revealing activity for Calcitriol at both 0.05 and 0.005 μM (Fig. 4C) and for Lovastatin at 0.25 μM (Fig. 4D). Tracer leakage assays validated significant permeability at 0.05 μM Calcitriol, but not 0.005 μM (Fig. 4E-F), indicating FishNAP’s greater sensitivity to subtle BBB changes. We also observed significantly increased permeability at 0.25 μM and 0.5 μM Lovastatin within 24 hours of treatment (Fig. 4E-F). Critically, repeating reversibility assays at 0.05 μM Calcitriol and 0.25 μM Lovastatin showed full recovery within 24 hours (Fig. 4G), confirming that all seven compounds reversibly modulate the BBB when administered at doses that minimize irreversible barrier damage.

### Assessing translational potential of BBB-opening small molecules

**Fig. 5.**
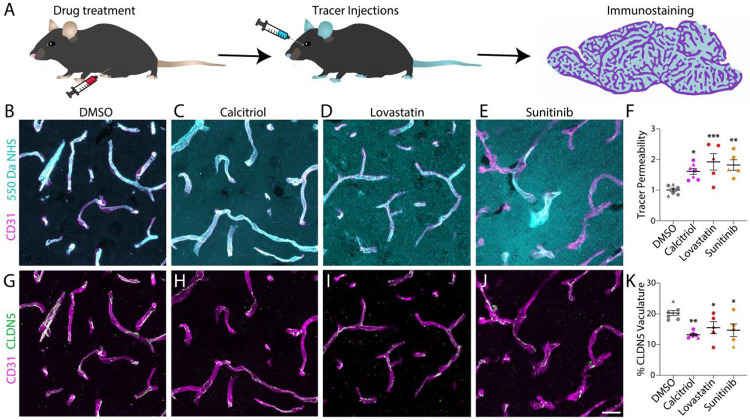
Small molecule treatment increases BBB permeability in adult mice. **A**. Schematic of drug treatment paradigm and downstream assays. **B-F**. Tracer leakage assays using 550 Da NHS-Biotin tracer (turquoise) in DMSO (B), Calcitriol (C), Lovastatin (D), and Sunitinib (E) treated mice reveals a significant increase in tracer permeability outside of the CD31+ vasculature (magenta) after drug treatment (F). **G-K**. Treatment with Calcitriol (H), Lovastatin (I), and Sunitinib (J) reduce endothelial expression of the tight junction CLDN5 (green) compared to DMSO controls (G), which maintain nice string like patterns and quantified in K. Values represent the average of 5 quantifications for an N of 7, 7, 5, and 5 mice for DMSO, Calcitriol, Lovastatin, and Sunitinib, respectively. Males are demarcated as triangles and females are demarcated as circles in F and K. * p<0.05 ** p<0.01 *** p<0.001 by one-way ANOVA. Scale bar represents 20 μm.

Finally, while our FishNAP assay enabled *in vivo* screening for BBB modulators in zebrafish (Fig. 2), it remained unclear whether these compounds would similarly affect the mammalian BBB. To test for cross-species conservation, we evaluated three of the BBB-opening small molecules identified by FishNAP that permeabilized the BBB to both sized tracers and acted through distinct signaling pathways—Calcitriol, Lovastatin, and Sunitinib—in adult mice. Mice were treated with each small molecule for two days prior to tracer leakage assays using the small 550 Da NHS-biotin tracer (Fig. 5A). While DMSO-treated controls confined the injected tracer within their CD31+ vasculature (Fig. 5B), Calcitriol-, Lovastatin-, and Sunitinib-treated mice exhibited significant increases in tracer leakage (Fig. 5C-F). To assess whether this enhanced BBB permeability arose from tight junction dysfunction, we immunostained for Claudin-5 (CLDN5) and observed a significant decrease in endothelial CLDN5 signal in treated mice (Fig. 5G-K), demonstrating that pathways uncovered in zebrafish translate directly to the mammalian BBB.

## Discussion

While zebrafish are a powerful model for studying BBB development and function^8,14,15,17,20^, most existing assays rely on invasive tracer injections or imaging-based approaches that are difficult to scale. Although these methods have yielded important insights, they are not well-suited for large-scale genetic or small-molecule screens. This is unfortunate, as zebrafish larvae readily absorb compounds directly from the water, making them highly tractable for *in vivo* pharmacology and chemical screening^16,21^. One persistent challenge in BBB drug development is the gap between *in vitro* activity and *in vivo* efficacy^22^. While many compounds modulate permeability *in vitro* BBB models, they frequently fail to penetrate the barrier *in vivo*^23^. *In vitro* systems are amenable to high-throughput screening but lack the cellular interactions, vascular architecture, hemodynamic forces, and whole-organism pharmacokinetics that shape barrier function. FishNAP addresses this gap by enabling rapid, nonlethal measurement of BBB permeability through a behavioral assay performed in a 96-well format in under an hour, without microinjection, confocal imaging, or extensive image analysis.

Using FishNAP, we screened a 2,320 compound FDA-approved library in under three months and identified several new molecular regulators of BBB function (Fig. 2), a level of speed and throughput not achievable with conventional tracer-based assays. In addition to chemical screening, FishNAP can assess BBB function in genetic mutants (Fig. 1). Because the assay is nonlethal and behavior-based, larvae displaying strong barrier phenotypes can be raised to establish stable lines with increased efficiency, making FishNAP well-suited for forward genetic screens that would be impractical in higher vertebrate models. Consistent with known BBB biology, FishNAP sensitively detects barrier changes across developmental and genetic contexts. The assay captures the transition from a permeable barrier at 3 dpf, when endothelial transcytosis is high, to a sealed barrier at 5 dpf as transcytosis is suppressed (Fig. 1B)^15^. It also robustly detects barrier defects in *spock1* mutants and *cldn5a* crispants, which model distinct disruptions in transcytosis regulation and tight junction organization (Fig. 1C & 1I)^8,18^. Together, these results demonstrate that FishNAP reliably reports changes in barrier integrity across diverse biological contexts.

The compounds identified in this screen map to diverse signaling pathways—including Hedgehog, vitamin D, farnesyltransferase, cholesterol synthesis, receptor tyrosine kinases, and MEK–ERK signaling—highlighting that BBB integrity can be modulated through multiple regulatory pathways rather than a single canonical mechanism. Notably, multiple small molecules targeting these pathways were present in the screened library, yet only a subset altered BBB permeability. This observation suggests that barrier modulation may depend on specific properties of individual compounds rather than pathway inhibition alone. For example, receptor tyrosine kinases comprise a large and diverse family, and it remains unclear whether the BBB-opening effects observed here reflect inhibition of particular signaling nodes, off-target activities, or structural features of the small molecules themselves. These findings therefore provide a starting point for future chemical and mechanistic studies aimed at defining the molecular determinants that enable selective modulation of BBB permeability.

FishNAP can generate apparent false-positive hits because it relies on a behavioral readout of decreased locomotion. Although highly sensitive to neuroactive compounds, this readout is not specific for BBB permeability and may reflect off-target drug effects or drug interactions. This likely explains one of our validated hits, the P-glycoprotein (Pgp) inhibitor Berbamine HCl, which consistently sedated fish after 24 h of treatment (Fig. 2). Because Pgp actively exports many hydrophobic compounds from the brain^24,25^, its inhibition likely increased loperamide accumulation in the brain, producing sedation without widespread barrier leakage. Similarly, the apparent hits observed with the EGFR inhibitors Afatinib dimaleate and Poziotinib may reflect enhanced sedation rather than increased BBB permeability, as fatigue is a known side effect of these therapies^26,27^. These findings underscore the importance of validating FishNAP hits with tracer-based assays to determine whether permeability into the brain truly increases and whether such changes exhibit size or regional restrictions^15^. Because these compounds are delivered systemically, multiple CNS barriers, including the arachnoid and blood–CSF barriers at the choroid plexus, may also be affected^28^. Neither FishNAP nor our tracer assays distinguish among these potential routes of entry. Defining which barriers are disrupted will require further mechanistic studies, including electron microscopy, long-term time-lapse imaging, transgenic permeability reporters, and single-cell transcriptomic profiling of endothelial and barrier-associated cells^8,15,17,29-31^.

Vitamin D signaling emerged as a potent regulator of BBB permeability in our screen, with both Calcifediol and Calcitriol reliably and reversibly increasing barrier permeability in zebrafish (Fig. 3 and 4). We further show that increased vitamin D signaling similarly modulates barrier function in the adult mouse brain (Fig. 5). These findings are somewhat unexpected given prior studies linking vitamin D deficiency to BBB dysfunction and suggesting that Calcitriol treatment can stabilize barrier integrity under chronic deficiency conditions^32-36^. Our results point to a more complex relationship between vitamin D signaling and BBB regulation, in which acute increases in vitamin D activity can transiently increase barrier permeability. Together, these observations suggest that the magnitude, duration, or cellular context of vitamin D signaling may influence whether barrier integrity is reinforced or transiently relaxed. More broadly, the diverse pathways uncovered in our screen suggest that BBB permeability can be modulated through multiple regulatory inputs rather than a single dominant pathway. Together, these findings position FishNAP as a platform for discovering and dissecting the signaling mechanisms that govern barrier permeability *in vivo*.

## Methods

### Zebrafish Lines and Maintenance

Zebrafish were maintained at 28.5°C following standard protocols^37^. All work was approved by the Rutgers University Standing Committee under protocol number 202300038. Adult fish were maintained on a standard 12 hour light-dark cycle. Adult fish, aged 3 months to 1.5 years, were crossed to produce embryos and larvae. For larvae used for confocal microscopy, 0.003% phenylthiourea (PTU; Sigma P7629) was applied beginning at 1 dpf to inhibit melanin production. These studies used the AB wild-type strain, the transgenic reporter strain Tg(*kdrl:HRAS-mCherry*)^s896Tg^ abbreviated as *kdrl:mCherry* in the text^38^, and the *spock1*^*hm43*^ BBB mutant line^8^. *Cldn5a* crispant fish were generated by injection of Cas9 protein and four guide RNAs (target sequences: GGACAACGTGAAAGCGCGGG, AGCGATGGCCTCCGCGGCTT, CAAGCACGAAGATGGCGCTG, GTGCGTCTGCGGGACGCTTT) into single-cell fertilized transgenic Tg(*kdrl:mCherry*) embryos in the AB background.

### FishNAP Assay

Fish were transferred to a 96 well square bottom plate, either individually or with 5-7 fish per well, and treated with 0.1 mM loperamide (Santa Cruz Biotechnology sc-203116) in 1x Danieau buffer immediately preceding the trial. The plate was then placed in a Daniovision recording chamber (Noldus). For grouped fish, mechanical tapping occurred every 10 seconds and lights flashed every 20 seconds. For individual fish, we used “rave” mode, with random tapping between 0-3 seconds and random light flashes every between 0-10 seconds to encourage activity. Larval locomotor activity was recorded for 1 hour in 10-minute bins and quantified using Ethovision XT13 software (Noldus). An activity ratio was calculated by dividing the average activity during the final 20 minutes by the average activity during the first 20 minutes. Each condition was normalized to same-day controls (wild-type, uninjected, or DMSO-treated) to minimize batch effects, and normalized activity values were then tested for statistical differences.

### Drug Screen

Wild-type zebrafish larvae were allocated into groups of seven per well in 24-well plates at 5 dpf. Larvae were exposed to 5 μM of two pooled compounds from the DiscoveryProbe^™^ FDA-approved Drug Library (APExBIO L1021) for two days and monitored for survival. At 7 dpf, larvae were analyzed using the FishNAP assay, with hits defined by an activity ratio < 0.6. Pooled hits or toxic pools were retested with individual compounds at 5 μM to identify specific active molecules. Compounds exhibiting toxicity at 5 μM were then retested at 1 μM and subsequently at 0.5 μM with persisting toxicity. Hit validation was then performed in multiple wells across at least two independent experiments, with activity compared to DMSO-treated controls for significance.

### Zebrafish Fluorescent Tracer Leakage Assays and Image Analysis

Zebrafish larvae were anesthetized with tricaine and positioned in an agarose injection mold. A 2.3 nL cocktail of Alexa 405 NHS Ester (10 mg/mL; Thermo Scientific A30000) and Alexa 647 10 kDa Dextran (5 mg/mL; Thermo Scientific D22914) was injected into the cardiac sac using a Nanoject III microinjector (Drummond Scientific). Larvae were then embedded in 1% low-melting-point agarose (Benchmark Scientific A1801-LM) and submerged in tricaine-containing embryo water for live imaging within 1 h post-injection. Imaging was performed on a Leica Stellaris DMi8 confocal microscope with a 25x water-immersion objective (0.75x zoom) and 1 μm z-steps. In FIJI^39^ a 20 μm maximum-intensity projection starting ~30 μm beneath the skin was used for analysis. Tracer intensity in the midbrain was quantified and normalized to intravascular tracer intensity for each fish. Representative images were made using 60 μm thick maximum-intensity projections.

### Mouse lines and manipulations

All mouse experiments were conducted in accordance with institutional and NIH guidelines and approved by the Rutgers University Standing Committee (protocol #202400110). Mice were housed under a 12 h light/12 h dark cycle. Wild-type C57BL/6J mice (Jackson Laboratory, strain 000664) aged 6–22 weeks, both male and female, were used. Calcitriol (1.2 μg g^− 1^ body weight; Sigma-Aldrich 32222-06-3) was administered intraperitoneally once daily for two days. On day 3, mice received a retro-orbital injection of EZ-Link sulfo-NHS-LC-biotin tracer (0.2 mg g^− 1^ body weight; Thermo Scientific 21335) under brief isoflurane anesthesia. Brains were collected 30 min later following rapid perfusion with 4% PFA, post-fixed overnight in methanol at 4°C, and subsequently processed for immunohistochemistry.

### Immunohistochemistry and Image Analysis

Dissected brain tissue was washed three times in PBS, equilibrated in 30% sucrose at 4°C, and flash-frozen in Tissue-Tek® O.C.T. compound (Sakura #4583) for cryosectioning at 25 μm thickness. Sagittal sections were blocked for 1 h at room temperature in PBS containing 10% normal donkey serum and 0.1% Triton X-100, then incubated overnight at 4°C with primary antibodies diluted in blocking buffer: goat anti-CD31 (1:100; R&D Systems AF3628), and rabbit anti-CLDN5 (1:100; Invitrogen 34-1600). Sections were subsequently washed in PBS and incubated for 2–3 h at room temperature with secondary antibodies (1:400; Jackson ImmunoResearch) and DyLight 405 Streptavidin (1:400; Jackson ImmunoResearch 016-470-084), then washed again in PBS and coverslipped.

Five cortical regions per mouse were imaged using a Leica Stellaris DMi8 confocal microscope with a 25× water objective (0.75x zoom). Maximum intensity projections were generated from 20 μm z-stacks and analyzed in FIJI for tracer permeability and CLDN5 coverage. Tracer permeability was calculated as the mean NHS intensity outside the vasculature normalized to that within vessels, with values normalized to DMSO-treated controls from the same day (a value of 1 indicates no leakage; >1 indicates increased leakage). CLDN5+ vasculature was quantified by thresholding CD31+ regions to define ROIs, then measuring the % area containing Huang-thresholded CLDN5 signal.

### Statistical Analysis

All statistical analyses were performed using Prism 10 (GraphPad Software). Two group comparisons were analyzed using an unpaired two-tailed t test. Multiple group comparisons were analyzed on the average values per fish with either one-way ANOVA, followed by a post hoc Tukey’s multiple comparison test. Sample size for all experiments was determined empirically using standards generally employed by the field, and no data was excluded when performing statistical analysis. ****p < 0.0001, ***p < 0.001, **p < 0.01, * p < 0.05 as stated in the Figure Legends. Standard error of the mean was calculated for all experiments and displayed as errors bars in graphs. Statistical details for specific experiments, including precision measures, statistical tests used, and definitions of significance can be found in the figure legends.

## Supplementary Material

Supplement 1
