# Supplementary material for: In vivo discovery of blood-brain barrier opening small molecules with FishNAP
Source: bioRxiv. 2026 Mar 20:2026.03.18.712473. Preprint. [Version 1] doi: 10.64898/2026.03.18.712473 (PMC13015469; doi:10.64898/2026.03.18.712473)
Supplement: Supplement 1 [file media-1.docx]

**Supplementary Information**


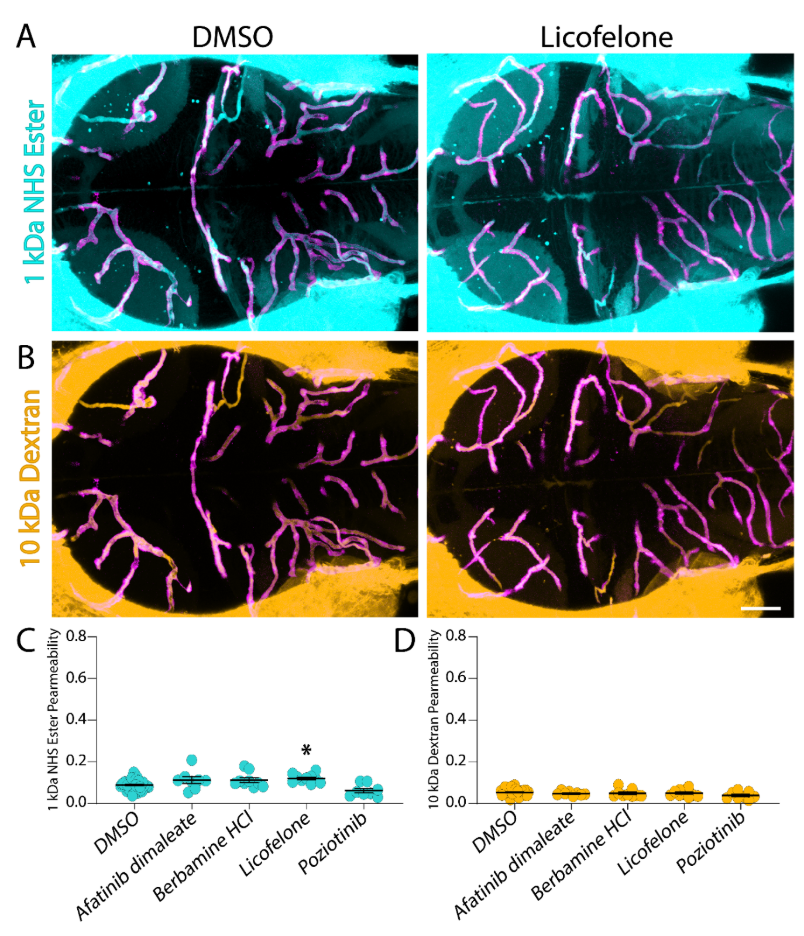


**Fig. S1. Four small molecules fail to cause substantial leakage of the BBB.** **A-B.** Functional tracer leakage assays with 1 kDa NHS Ester (turquoise, A) and 10 kDa Dextran (gold, B) reveal a small increase in 1 kDa NHS leakage after Licofelone treatment. **C-D.** Quantification of leakage in all treated fish for 1 kDa NHS Ester (C) and 10 kDa Dextran (D). Each point represents an individual fish. * p<0.05 by one-way ANOVA compared to DMSO controls. Scale bar represents 50 µm.
